# Treatment for a B-cell acute lymphoblastic leukemia patient carrying a rare *TP53* c.C275T mutation: A case report

**DOI:** 10.3389/fonc.2022.1018250

**Published:** 2023-01-31

**Authors:** Runan Wang, Wenliang Wang, Xuan Liu, Huan Wang, Bin Zhang, Shuang Li, Haining Zhang, Jiawei Yang, Jishun Zhao, Qiuying He, Jihong Zhang, Danping Liu, Liangchun Hao

**Affiliations:** ^1^ Department of Pediatrics, Shengjing Hospital of China Medical University, Shengyang, Liangning, China; ^2^ Precision Targeted Therapy Discovery Center, Institute of Technology Innovation, Hefei Institutes of Physical Science, Chinese Academy of Sciences, Hefei, Anhui, China; ^3^ Hematology Laboratory, Shengjing Hospital of China Medical University, Shengyang, Liangning, China

**Keywords:** B-cell acute lymphoblastic leukemia, TP53 c.C275T mutation, bortezomib, MRD, children

## Abstract

*TP53* mutations are associated with poor prognosis in the vast majority of cancers. In this study, we present a pediatric B-cell acute lymphoblastic leukemia (B-ALL) patient carrying a rare *TP53* c.C275T mutation. This extremely rare mutation affects an amino acid residue located between the TAD domain and the DNA-binding domain of p53. The patient was resistant to most conventional chemotherapy regimens and remained minimal residual disease (MRD)-positive after five rounds of such regimens. We tested the sensitivity of the patient’s leukemic cells to 21 anti-cancer drugs by performing *in vitro* drug sensitivity assays. The results showed that bortezomib had a very strong killing effect on the patient’s leukemic cells. Therefore, we subsequently treated the patient with bortezomib combined with vindesine, cytarabine, and fludarabine. After one course of treatment, the patient became MRD-negative, and there was no recurrence during a 9-month follow-up. In conclusion, our report suggests that the *TP53* c.C275T mutation is associated with poor prognosis in B-ALL. Fortunately, bortezomib combined with chemotherapy could achieve a better therapeutic effect than conventional regimens in this type of ALL.

## Introduction

B-cell acute lymphoblastic leukemia (B-ALL) is a type of ALL caused by malignant transformation and cloning of B-cell precursors in the bone marrow and thymus (1–3). Patients with B-ALL usually present with symptoms such as infection, anemia, hemorrhage, and tissue infiltration, which arise owing to the destruction of normal hematopoietic function and the accumulation of tumor cells (4–6).

B-ALL is the most common type of ALL in children, and nearly 20% of B-ALL patients relapse and die from the disease (7, 8). Children with relapsed B-ALL have poor prognosis, with overall survival rates as low as 35% to 40% even after intensive chemotherapy or stem cell transplantation (9, 10). Minimal residual disease (MRD) is an important factor leading to chemotherapy resistance and tumor recurrence in B-ALL. Targeted treatment has shown improved efficacy in MRD-positive pediatric B-ALL patients (11, 12).

Genetic alterations affecting genes involved in cell proliferation, cell differentiation, and apoptosis have been implicated in ALL. These genetic alterations include chromosomal rearrangements, chromosomal gains or losses, deletions, and point mutations (13, 14). Only 6% to 8% of *TP53* genetic alterations have been identified in ALL patients (15). These *TP53* mutations were mainly identified in relapsed lesions of pediatric ALL patients and were associated with poor therapy responses. Common mutations in *TP53* include R175H, H179R, H193R, V216M, G245S, and R273C. Although the roles of these common mutations in tumors have been extensively studied and reported (16–19), the role of rare *TP53* mutations in ALL has not been thoroughly investigated.

Herein, we present a case report of a pediatric B-ALL patient carrying a rare *TP53* c.C275T mutation. We summarize the clinical features of this patient and the efficacy of our treatment regimens, thereby providing a reference for the diagnosis and management of pediatric B-ALL patients carrying this mutation.

## Case presentation

**Figure 1:**
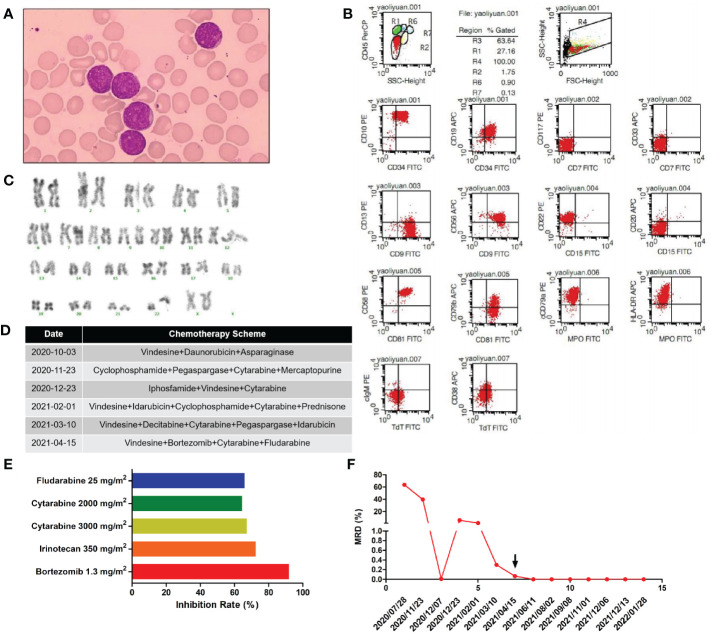
**(A)** Morphology of the patient’s lymphoblasts (scale: 10×). **(B)** Results of our flow cytometry analysis. **(C)** Karyotype analysis for this patient. **(D)** Treatment flow chart. **(E)** Sensitivity of the patient’s leukemic cells to 21 drugs tested. Drugs with a relative inhibition rate higher than 50% are listed. **(F)** MRD values during the treatment period.

A 2-year-old girl was admitted to our hospital on July 28, 2020, owing to persistent lymph node enlargement for 3 months. She had no family history of genetic disorders. Laboratory evaluation demonstrated a white blood cell count of 5.39×10^9^/L, a hemoglobin level of 92 g/L, a platelet count of 90×10^9^/L, a neutrophil ratio of 0.8%, and a lymphocyte ratio of 89.6%. Notably, immature cells accounted for 85% of peripheral blood cells. Bone marrow aspiration was performed and revealed hypercellularity with predominant blasts, which was in accordance with the bone marrow findings of L2-type ALL (**Figure 1A**). Flow cytometry showed that the blasts were mainly positive for CD45min, CD34, CD10, CD19, CD9, CD56, CD22, CD58, CD81, cCD79a, and HLA-DR, partially positive for CD79b and cTdT, and negative for CD7, CD117, CD33, CD13, CD20, CD15, CD38, cIgM, and MPO, indicating a diagnosis of early-stage B-ALL (**Figure 1B**). Karyotyping analysis of the peripheral blood illustrated that the patient had normal karyotype (**Figure 1C**). Reverse transcription PCR covering the 72 commonly detected fusion genes in leukemia was performed on the bone marrow sample and detected no gene fusion (**Supplementary Table 1**). Next-generation sequencing covering the 236 commonly mutated genes in ALL was performed and identified a *KDM6B* (NM_001080424) mutation: exon 22: c.C5042A (p.S1681X) (48.31%) and a *TP53* (NM_000546) mutation: exon 4: c.C275T (p.P92L) (50.46%) (**Supplementary Table 2**).

Chemotherapy schemes received by the patient are shown in **Figure 1D**. On day 30 after the first induction, there were still 39.6% blasts in the bone marrow as revealed by flow cytometry, suggesting resistance to routine chemotherapy. For economic reasons, family members refused bone marrow transplantation and CAR-T therapy and insisted on chemotherapy despite persistent MRD positivity. Then, we modified the induction chemotherapy regimen. At the same time, a second course of induction therapy was given to the patient. However, by day 30 after the second induction, the patient had still failed to achieve complete remission (CR), with 5.7% blasts in the bone marrow. Afterwards, the patient received three additional rounds of induction therapy but was still MRD-positive, as detailed in **Figure 1F**. Overall, the patient was failed to achieve CR after five rounds of induction therapy. Then, leukemic cells were obtained from bone marrow aspirates of the patient by Ficoll density gradient centrifugation and cultured in ALL complete medium (Precedo, Hefei, China) for drug sensitivity analysis of a panel of 21 anti-cancer drugs, including targeted therapy drugs, and antibody drugs (the 21 anti-cancer drugs are listed in **Supplementary Table 3**). The drug concentrations in the *in vitro* experiment were converted according to the dose and Css data from clinical trials. The results showed that the patient’s leukemic cells were resistant to most of the drugs commonly used in ALL treatment, which was consistent with our *in vivo* results (**Supplementary Table 3**). Drugs with an inhibition rate greater than 50% are shown in **Figure 1E**. Finally, the patient received the scheme vincristine 1.5 mg/m^2^ day 1 + cytarabine 2000 mg/m^2^ days 1-3 + bortezomib 1 mg/m^2^ days 1 and 5, + fludarabine 25 mg/m^2^ days 1 -5, and achieved CR and became MRD-negative. Then, two cycles of the same scheme were given as consolidation chemotherapies. Owing to personal reasons, the patient did not receive a subsequent bone marrow transplantation and was discharged from the hospital for maintenance treatment (vincristine 1.5 mg/m^2^ day 1 + dexamethasone 8 mg/m^2^ days 1 - 5 + methotrexate 25 mg/m^2^ days 8, 15 and 22 + mercaptopurine 50 mg/m^2^ days 8-28) and was followed up regularly every 1-2 months. The patient remained leukemia-free and MRD-negative at 9-month follow-up and was lost to follow-up thereafter.

## Discussion

ALL is the most common type of childhood cancer worldwide and chemotherapy is the main treatment for this malignancy. Although the current 5-year survival rate of children with ALL is as high as 80%-90%, 15%-20% of children with ALL will experience relapse (20, 21). After recurrence, the 5-year survival rate is only 30%-40%. Drug resistance-associated recurrence is an important cause of treatment failure in pediatric ALL (22–24). Deciphering the mechanisms of drug resistance and relapse in pediatric ALL and optimizing the current treatment plans have become the focus of research in this field.

*TP53* has long been a “star” gene in the field of cancer research. About half of malignant tumor types are associated with *TP53* mutations (25). There are various types of *TP53* gene mutations, including deletions, insertions, and missense point mutations. Among them, missense point mutations account for up to 80% of all mutations. There are two mechanisms by which point mutations affect the interaction between p53 and DNA. One is that certain p53 mutations, such as those affecting residues Arg248 and Arg273, impede the contact between p53 and DNA. The other is that some mutations (such as those affecting Arg175, Gly245, Arg249, and Arg282 residues) of p53 prevent the protein from folding properly and binding tightly to DNA, thereby destroying the protein’s tumor suppressor ability. p53 mutants not only lose their tumor-suppressing activity but also have disrupted the function of wild-type p53 proteins, which further promotes the progression of tumors (26–28). For example, myelodysplastic syndrome and acute myeloid leukemia patients with *TP53* mutations were found to have poor chemotherapy response and short remission period, especially those with biallelic mutations of *TP53* and those with complex karyotypes, who were more prone to relapse even after bone marrow transplantation (29, 30).

In ALL, *TP53* is altered at a frequency of 19% and mutated at a frequency of only 8% (15). In relapsed ALL, however, the *TP53* mutation frequency rises to about 10% and represents a strong and independent predictor of treatment failure. Around 80% of *TP53* point mutations affect the DNA-binding domain of the protein. In this study, we identified a c.C275T mutation in *TP53*. This mutation converts Pro 92, an amino acid residue located between the TAD domain and the DNA-binding domain of p53, into leucine. Although we did not investigate the effect of this mutation on the function of the p53 protein, we found that our B-ALL patient with this mutation had severe disease and had failed to fully respond to most clinical treatments. So far, there are two reports in the literature of *TP53* c.C275T mutation. The first report describes a study of actinic keratosis. The researchers identified *TP53* c.C275T mutation in a patient with actinic keratosis (31). The other report describes a study of chronic lymphocytic leukemia (CLL). The researchers found that the frequency of *TP53* mutations was significantly higher in CLL patients in Taiwan than in the West. The *TP53* c.C275T mutation was detected in one patient. However, the role of *TP53* c.C275T mutation in disease initiation and progression has not been investigated (32). We also found that our patient carried a *KDM6B* c.C5042A mutation, although this was a stop-gain mutation. However, compared with the wild-type KDM6B, the mutant protein had only lost two amino acids at the end of the C-terminus. Protein secondary structure prediction analysis showed no known functional motif at the extreme C-terminal end, thus, it is unlikely that this mutation affects the overall protein conformation and function. Therefore, we speculate that *TP53* c.C275T mutations in this patient are predominantly.

Bortezomib is a reversible inhibitor of the chymotrypsin-like activity of the 26S proteasome in mammalian cells. *In vitro* tests have demonstrated that bortezomib is toxic to various types of cancer cells. *In vivo* studies in preclinical tumor models have demonstrated that bortezomib delays the growth of tumors of various types, including multiple myeloma (33, 34). Bortezomib has been approved as a single agent for the treatment of previously untreated multiple myeloma patients who are not suitable for high-dose chemotherapy and myelosuppression or who have relapsed after at least one course of treatment (35–37). Owing to its excellent therapeutic effect against multiple myeloma, the use of bortezomib has been actively expanded into the treatment of other cancers. A number of clinical studies of bortezomib in ALL are currently underway. One study found that bortezomib monotherapy was not an ideal treatment for ALL because no significant clinical response was observed (38). On the other hand, bortezomib combined with other chemotherapy regimens exhibited a significantly improved treatment effect in ALL patients. A phase II clinical trial found that 73.6% of patients with relapsed ALL achieved CR after receiving bortezomib combined with chemotherapy (39). Another study showed that 88.9% of patients with relapsed or refractory ALL achieved CR after receiving bortezomib combined with chemotherapy (40). A study conducted by Bertaina et al. demonstrated that combination of bortezomib with chemotherapy achieved a remarkable effect in relapsed/refractory ALL of childhood. Twenty-seven patients (72.9%) achieved CR or CR with incomplete platelet recovery (CRp). Twenty-two of 30 BCP-ALL patients (73.3%) and five of seven patients (71%) with T-cell ALL achieved CR/CRp (41). Hasegawa et al. reported that CR was achieved in all three patients with a combination of bortezomib and chemotherapy (42). A phase III clinical trial (AALL1231) conducted by Teachey et al. demonstrated that outcomes for SR and IR T-ALL patients treated with bortezomib were excellent despite elimination of prophylactic CRT (43).

In our study, the B-ALL patient with a *TP53* c.C275T mutation remained MRD-positive after multiple rounds of conventional chemotherapy. Subsequently, we found *via* high-throughput drug sensitivity tests that bortezomib had a strong killing effect on the patient’s leukemic cells. Therefore, we treated the patient with bortezomib combined with chemotherapy and found that the patient became MRD-negative after one round of the treatment. In addition, no recurrence was observed during the subsequent 9-month follow-up. It has been reported that bortezomib could induce apoptosis by activating caspase-3 activity in p53-deficient cells (44). Therefore, we speculated that bortezomib may have inhibited ALL cells with *TP53* c.C275T mutation through a similar mechanism.

In conclusion, our study suggests that the *TP53* c.C275TC mutation is indicative of poor prognosis in ALL. Fortunately, bortezomib combined with chemotherapy achieved a good therapeutic effect in our patient. This study is expected to provide new ideas for the treatment of ALL patients with the *TP53* c.C275T mutation.

## Data availability statement

The datasets presented in this article are not readily available because of ethical/privacy restrictions. Requests to access the datasets should be directed to the corresponding author.

## Ethics statement

Ethical approval was not provided for this study on human participants because Ethical review and approval was not required for this study in accordance with the local legislation and institutional requirements. Written informed consent to participate in this study was provided by the participants’ legal guardian/next of kin. Written informed consent was obtained from the individual(s), and minor(s)’ legal guardian/next of kin, for the publication of any potentially identifiable images or data included in this article.

## Author contributions

Funding acquisition: RW, Investigation: RW, XL, JZhao, Resources: HW, QH, Data curation: BZ, SL, HZ, JY, JZhang, DL, Formal analysis: WW, Project administration: LH, Writing-review & editing: WW, Supervision: LH, RW, WW.

## References

[1] Baruchel A Schaison G . Recent advances in b cell acute lymphoblastic leukemia (Burkitt leukemia) therapy in childhood. Nouv Rev Fr Hematol (1993) 35(1):106–8. doi: 10.1017/cbo9780511977633.016 8511033

[2] Rosanda C Cantù-Rajnoldi A Invernizzi R Castagni M Cataldo A Fenu S . B-cell acute lymphoblastic leukemia (B-ALL): a report of 17 pediatric cases. Haematologica (1992) 77(2):151–5. doi: 10.1007/978-981-15-0548-5_15 1398300

[3] Lalit S Rajesh K Sunil G Bhargava M . B-cell acute lymphoblastic leukemia in a child with ataxia telangiectasia. Pediatr Hematol Oncol (2008) 25(5):473–6. doi: 10.1080/08880010802106614 18569850

[4] Lowichik A Bernini J Tonk V Ansari M Rollins N Winick N . Relapse of precursor b-cell acute lymphoblastic leukemia as an isolated central nervous system mass lesion 9 years after initial diagnosis. Med Pediatr Oncol (1996) 26(2):129–34. doi: 10.1200/jco.1985.3.5.622 8531851

[5] Wang J Huang F Chi C Chou G Chang T . Skull mass as a heralding sign of precursor b-cell acute lymphoblastic leukemia in a toddler. J Pediatr (2006) 149(4):577. doi: 10.1016/j.jpeds.2006.05.049 17011339

[6] Salaverria I Siebert R . The gray zone between burkitt's lymphoma and diffuse large b-cell lymphoma from a genetics perspective. J Clin Oncol (2011) 29(14):1835–43. doi: 10.1200/JCO.2010.32.8385 21482997

[7] Fujita N Kobayashi R Atsuta Y Iwasaki F Suzumiya J Sasahara Y . Hematopoietic stem cell transplantation in children and adolescents with relapsed or refractory b-cell non-Hodgkin lymphoma. Int J Hematol (2019) 109(4):483–90. doi: 10.1007/s12185-019-02608-y 30701466

[8] Jeffrey W Konrad MA Ge Y Naber JM Scott JS Matherly LH . High frequency of leukemic clones in newborn screening blood samples of children with b-precursor acute lymphoblastic leukemia. Blood (2002) 99(8):2992–6. doi: 10.1182/blood.v99.8.2992 11929791

[9] Shukla N Sulis ML . Blinatumomab for treatment of children with high-risk relapsed b-cell acute lymphoblastic leukemia. JAMA (2021) 325(9):830–2. doi: 10.1001/jama.2021.1395 33651075

[10] Asare JM Rabik CA Muller B Brown PA Cooper S . Investigational treatment options in phase I and phase II trials for relapsed or refractory acute lymphoblastic leukemia in pediatric patients. Expert Opin Investig Drugs (2021) 30(6):611–20. doi: 10.1080/13543784.2021.1916466 33896328

[11] Kruse A Abdel-Azim N Kim HN Ruan Y Phan V Ogana H . Minimal residual disease detection in acute lymphoblastic leukemia. Int J Mol Sci (2020) 21(3):1054. doi: 10.3390/ijms21031054 32033444 PMC7037356

[12] Medinger M Heim D Lengerke C Halter J Passweg J . Acute lymphoblastic leukemia diagnosis and therapy. Ther Umsch (2019) 76(9):510–5. doi: 10.1024/0040-5930/a001127 32157966

[13] Inaba H Mullighan C . Pediatric acute lymphoblastic leukemia. Haematologica (2020) 105(11):2524–39. doi: 10.3324/haematol.2020.247031 PMC7604619 33054110

[14] Terwilliger T Abdul-Ha M . Acute lymphoblastic leukemia: a comprehensive review and 2017 update. Blood Cancer J (2017) 7(6):e577. doi: 10.1038/bcj.2017.53 28665419 PMC5520400

[15] Salmoiraghi S Rambaldi A Spinelli O . TP53 in adult acute lymphoblastic leukemia. Leuk Lymphoma (2018) 59(4):778–89. doi: 10.1080/10428194.2017.1344839 28679301

[16] Comeaux E Mullighan C . TP53 mutations in hypodiploid acute lymphoblastic leukemia. Cold Spring Harb Perspect Med (2017) 7(3):a026286. doi: 10.1101/cshperspect.a026286 28003275 PMC5334249

[17] Yu C Chang W Jou S Lin T Chang Y Lin C . TP53 alterations in relapsed childhood acute lymphoblastic leukemia. Cancer Sci (2020) 111(1):229–38. doi: 10.1111/cas.14238 PMC6942420 31729120

[18] Ueno H Yoshida K Shiozawa Y Nannya Y Iijima-Yamashita Y Kiyokawa N . Landscape of driver mutations and their clinical impacts in pediatric b-cell precursor acute lymphoblastic leukemia. Blood Adv (2020) 4(20):5165–73. doi: 10.1182/bloodadvances.2019001307 PMC7594377 33095873

[19] Demir S Boldrin E Sun Q Hampp S Tausch E Eckert C . Therapeutic targeting of mutant p53 in pediatric acute lymphoblastic leukemia. Haematologica (2020) 105(1):170–81. doi: 10.3324/haematol.2018.199364 PMC6939517 31073076

[20] Inaba H Pui C . Immunotherapy in pediatric acute lymphoblastic leukemia. Cancer Metastasis Rev (2019) 38(4):595–610. doi: 10.1007/s10555-019-09834-0 31811553 PMC6995750

[21] Abdelmabood S Fouda A Boujettif F Mansour A . Treatment outcomes of children with acute lymphoblastic leukemia in a middle-income developing country: high mortalities, early relapses, and poor survival. J Pediatr (Rio J) (2020) 96(1):108–16. doi: 10.1016/j.jped.2018.07.013 PMC9432263 30240631

[22] Ma J Chen Y Yu L . Research progress on drug-resistance of acute lymphoblastic leukemia–review. Zhongguo Shi Yan Xue Ye Xue Za Zhi (2016) 24(1):261–5. doi: 10.20517/cdr.2019.11 26913433

[23] Chen S . Asparaginase therapy in pediatric acute lymphoblastic leukemia: A focus on the mode of drug resistance. Pediatr Neonatol (2015) 56(5):287–93. doi: 10.1016/j.pedneo.2014.10.006 25603726

[24] Pogorzala M Kubicka M Rafinska B Wysocki M Styczynski J . Drug-resistance profile in multiple-relapsed childhood acute lymphoblastic leukemia. Anticancer Res (2015) 35(10):5667–70. doi: 10.1007/978-3-319-39708-5_11 26408741

[25] Aubrey B Strasser A Kelly G . Tumor-suppressor functions of the TP53 pathway. Cold Spring Harb Perspect Med (2016) 6(5):a026062. doi: 10.1385/1-59259-328-3:117 27141080 PMC4852799

[26] Petitjean A Mathe E Kato S Ishioka C Tavtigian S Hainaut P . Impact of mutant p53 functional properties on TP53 mutation patterns and tumor phenotype: lessons from recent developments in the IARC TP53 database. Hum Mutat (2007) 28(6):622–9. doi: 10.1002/humu.20495 17311302

[27] Bykov V Eriksson S Bianchi J Wiman K . Targeting mutant p53 for efficient cancer therapy. Nat Rev Cancer (2018) 18(2):89–102. doi: 10.1038/nrc.2017.109 29242642

[28] Li L Li M Wang X . Cancer type-dependent correlations between TP53 mutations and antitumor immunity. DNA Repair (Amst) (2020) 88:102785. doi: 10.1016/j.dnarep.2020.102785 32007736

[29] Sallman D DeZern A Garcia-Manero G Steensma D Roboz G Sekeres M . Eprenetapopt (APR-246) and azacitidine in TP53-mutant myelodysplastic syndromes. J Clin Oncol (2021) 39(14):1584–94. doi: 10.1200/JCO.20.02341 PMC8099410 33449813

[30] Niparuck P Police P Noikongdee P Siriputtanapong K Limsuwanachot N RerkamnuaychokeB . TP53 mutation in newly diagnosed acute myeloid leukemia and myelodysplastic syndrome. . Diagn Pathol (2021) 16(1):100. doi: 10.1186/s13000-021-01162-8 34717674 PMC8557522

[31] Ziegler A Jonason AS Leffell DJ Simon JA Sharma WH Kimmelman J . Sunburn and p53 in the onset of skin cancer. Nature (1994) 372(6508):773–6. doi: 10.1038/372773a0 7997263

[32] Wu SJ Lin CT Agathangelidis A Lin LL Kuo YY Tien HF . Distinct molecular genetics of chronic lymphocytic leukemia in Taiwan: clinical and pathogenetic implications. Haematologica (2017) 102(6):1085–90. doi: 10.3324/haematol.2016.157552 PMC5451340 28255015

[33] Scott K Hayden P Will A Wheatley K Coyne K . Bortezomib for the treatment of multiple myeloma. Cochrane Database Syst Rev (2016) 4:CD010816. doi: 10.1007/978-3-7643-8948-2_4 27096326 PMC10387344

[34] Robak P Robak T . Bortezomib for the treatment of hematologic malignancies: 15 years later. Drugs R D (2019) 19(2):73–92. doi: 10.1007/s40268-019-0269-9 30993606 PMC6544598

[35] Breitkreutz I Raab M Goldschmidt H . First-line treatment of multiple myeloma. Internist (Berl) (2019) 60(1):23–33. doi: 10.1007/s00108-018-0527-x 30552458

[36] Kropff M Bisping G Schuck E Liebisch P Lang N Hentrich M . Bortezomib in combination with intermediate-dose dexamethasone and continuous low-dose oral cyclophosphamide for relapsed multiple myeloma. Br J Haematol (2007) 138(3):330–7. doi: 10.1111/j.1365-2141.2007.06656.x 17614819

[37] Wang Y Ding S Wu F Wang Z Wang Q . Safety and efficacy of subcutaneous administration of bortezomib in the treatment of multiple myeloma. Sichuan Da Xue Xue Bao Yi Xue Ban (2014) 45(3):529–32. doi: 10.47939/mh.v2i11.402 24941833

[38] Cortes J Thomas D Koller C . Phase I study of bortezomib in refractory or relapsed acute leukemias. Clin Cancer Res (2004) 10(10):3371–6. doi: 10.1158/1078-0432.ccr-10-22-corb 15161691

[39] Messinger YH Gaynon PS Sposto R . Bortezomib with chemotherapy is highly active in advanced b-precursor acute lymphoblastic leukemia: Therapeutic advances in childhood. Leukemia Lymphoma (TACL) Study. Blood (2012) 120(2):285–90. doi: 10.3410/f.717957011.793461504 22653976

[40] Zhao J Wang C Song Y Liu Y Fang B . Treatment of refractory/relapsed adult acute lymphoblastic leukemia with bortezomibbased chemotherapy. Int J Gen Med (2015) 8:211–4. doi: 10.2147/ijgm.s59537 PMC4472074 26109875

[41] Bertaina A Vinti L Strocchio L Gaspari S Caruso R Algeri M . The combination of bortezomib with chemotherapy to treat relapsed/refractory acute lymphoblastic leukaemia of childhood. Br J Haematol (2017) 176(4):629–36. doi: 10.1111/bjh.14505 28116786

[42] Hasegawa D Yoshimoto Y Kimura S Kumamoto T Maeda N Hara J . Bortezomib-containing therapy in Japanese children with relapsed acute lymphoblastic leukemia. Int J Hematol (2019) 110(5):627–34. doi: 10.1007/s12185-019-02714-x 31401767

[43] Wu SJ Lin CT Agathangelidis A Lin LI Kuo YY Tien HF . Distinct molecular genetics of chronic lymphocytic leukemia in Taiwan: clinical and pathogenetic implications. J Clin Oncol (2022) 40(19):2106–18. doi: 10.1200/JCO.21.02678 PMC5451340 28255015

[44] Yerlikaya A Okur E Ulukaya E . The p53-independent induction of apoptosis in breast cancer cells in response to proteasome inhibitor bortezomib. Tumour Biol (2012) 33(5):1385–92. doi: 10.1007/s13277-012-0386-3 22477712

