# Supplementary material for: Treatment for a B-cell acute lymphoblastic leukemia patient carrying a rare TP53 c.C275T mutation: A case report
Source: Front Oncol. 2023 Jan 31;12:1018250. doi: 10.3389/fonc.2022.1018250 (PMC9928200; doi:10.3389/fonc.2022.1018250)
Supplement: Supplementary file 1 [file DataSheet_1.pdf]

Supplementary table 1: 72 common fusion genes in leukemia were detected with RT-PCR assay

BCR-ABL	*TEL-PDGFRB*	*SIL-TAL1*	*FIP1L1-PDGFRA*	*E2A-HLF*	*AML1-MDS1*
AML1-MTG16	*TEL-AML1*	*CBFB-MYH11*	*MLL-AF4*	*DEK-CAN*	*E2A-PBX1*
NPM-MLF1	*AML1-ETO*	*TEL-ABL*	*MLL-AF9*	*ETV6-PDGFRA*	*PML-RARA*
NUP98-HOXA13	*NUP98-HOXC11*	*NUP98-HOXD13*	*NUP98-HOXA9*	*NUP98-HOXA11*	*NUP98-PMX1*
MLL-AF6	*MLL-AF10*	*MLL-ELL*	*MLL-ENL*	*MLL-AF17*	*MLL-AF1q*
MLL-AF1P	*MLL-AFX*	*MLL-SEPT6*	*PLZF-RARA*	*STATB-RARA*	*TEL-JAK2*
NPM-RARA	*FIP1L1-RARA*	*PRKAR1A-RARA*	*NUMA1-RARA*	*NPM-ALK*	*TLS-ERG*
SET-CAN	*ETV6-ABL1*	*RCSD1-ABL1*	*RSCD1-ABL2*	*MYB-TYK*	*NUP214-ABL1*
MYH9-IL2RB	*PAX5-JAK2*	*CRLF2*	*IKZF1*	*PAG1-ABL2*	*SSBP2-JAK2*
ZMIZ1-ABL1	*STRN3-JAK2*	*TERF2-JAK2*	*SSBP2-PDGFRB*	*TNIP1-PDGFRB*	*ZEB2-PDGFRB*
BCR-JAK2	*EBF1-JAK2*	*SSBP2-CSF1R*	*ETV6-JAK2*	*PPFIBP1-JAK2*	*SNX2-ABL1*
ETV6-NTRK3	*ZC3HAV1-ABL2*	*EBF1-PDGFRB*	*TPR-JAK2*	*RANBP2-ABL1*	*ATF7IP-JAK2*

Supplementary table 2: 236 commonly mutated genes in leukemia were detected with Next generation sequencing assay

ABL1	*ANKRD26*	*ARID1B*	*ARID2*	*ASXL1*	*ASXL2*
ATM	*B2M*	*BCL2*	*BCL6*	*BCOR*	*BCORL1*
BIRC3	*BLM*	*BRAF*	*BTK*	*CALR*	*CARD11*
CBL	*CCND1*	*CD28*	*CD58*	*CD79A*	*CD79B*
CDKN1B	*CDKN2A*	*CEBPA*	*CHD8*	*CREBBP*	*CSF3R*
CXCR4	*DDX41*	*DHX15*	*DKC1*	*DNMT3A*	*ELANE*
EP300	*EPOR*	*ETNK1*	*ETV6*	*EZH2*	*FBXW7*
FLT3	*FOXO1*	*GATA1*	*GATA2*	*GATA3*	*GFI1*
GNA13	*HAX1*	*ID3*	*IDH1*	*IDH2*	*IKZF1*
IL7R	*JAK1*	*JAK2*	*JAK3*	*KDM6A*	*KIT*
KMT2A	*KMT2C*	*KMT2D*	*KRAS*	*MAP2K1*	*MAPK1*
MEF2B	*MLH1*	*MPL*	*MSH6*	*MYD88*	*NF1*
NOTCH1	*NOTCH2*	*NPM1*	*NRAS*	*NTRK3*	*PAX5*
PDGFRB	*PHF6*	*PIGA*	*PLCG2*	*POT1*	*PPM1D*
PRPF8	*PTEN*	*PTPN11*	*RAD21*	*RB1*	*RHOA*
RUNX1	*SBDS*	*SETBP1*	*SF3B1*	*SH2B3*	*SMC1A*
SMC3	*SRP72*	*SRSF2*	*STAG2*	*STAT3*	*STAT5B*
TCF3	*TERC*	*TERT*	*TET2*	*TNFAIP3*	*TNFRSF14*
TP53	*TPMT*	*TRAF3*	*U2AF1*	*WT1*	*ZRSR2*
ABCB1	*ABCC3*	*ABCG2*	*ADSL*	*AKT2*	*AKT3*
ALK	*AMER1*	*ATRX*	*BCL11B*	*BMP7*	*CACNA1E*
CACNA1G	*CCDC168*	*CCND3*	*CDA*	*CECR2*	*CEP72*

CHD2	*CPA2*	*CRLF2*	*CSMD1*	*CTCF*	*CTLA4*
CTNNB1	*CUX1*	*CYBA*	*CYP2B6*	*CYP2C19*	*CYP2C8*
CYP3A4	*CYP3A5*	*DARS*	*DCTD*	*DHX30*	*DIS3*
DNAH2	*DNM2*	*DOK5*	*DROSHA*	*DYNC2H1*	*EGR2*
ERCC1	*ERG*	*EVI1*	*FAM46C*	*FAT1*	*FCGR3A*
FGFR1	*GNAS*	*GSTM1*	*GSTP1*	*HIST1H1E*	*HLA-DRB1*
IL2RB	*IMPDH2*	*IRF4*	*ITPA*	*KDM5C*	*KDM6B*
LINC00251	*MACF1*	*MAP3K7*	*MED12*	*MTHFR*	*MTRR*
MYC	*NF2*	*NFATC2*	*NFKBIA*	*NFKBIE*	*NR3C1*
NSD2	*NT5C2*	*NTRK1*	*NUDT15*	*PCLO*	*PDGFRA*
PIK3CA	*PIK3R1*	*PNPLA3*	*PRKDC*	*PROX1-AS1*	*PRPS1*
RIT1	*ROBO1*	*ROBO2*	*ROBO3*	*RPL10*	*RPS15*
RRM1	*RRM2*	*RRM2B*	*SAMHD1*	*SERPINE1*	*SETD2*
SF1	*SLC22A1*	*SLC29A1*	*SLCO1A2*	*SLCO1B1*	*SMAD4*
SOCS1	*SOD2*	*SOS1*	*SOX11*	*SPI1*	*SRCAP*
STAG1	*STAT5A*	*STAT6*	*STIM1*	*SUZ12*	*TNF*
TRIM24	*UGT1A1*	*UGT1A8*	*USH2A*	*USP7*	*XPO1*
XRCC5	*ZMYM3*				

Supplementary table 3: The result of in vitro drug sensitivity screening

**Drugs**	**Classification**	**Dosage**	**Inhibition rate (%)**
Bortezomib	molecular targeted therapeutic drugs	1.3 mg/m^2	91.93
Irinotecan	chemotherapy regimens	350 mg/m^2	72.55
Cytarabine	chemotherapy regimens	3000 mg/m^2	67.18
		2000 mg/m^2	64.41
		100 mg/m^2	33.27
Fludarabine	chemotherapy regimens	25 mg/m^2	66.02
Etoposide	chemotherapy regimens	100 mg/m^2	49.15
		60 mg/m^2	32.46
Cladribine	chemotherapy regimens	0.09 mg/kg	43.21
Asparaginase	other	10000U	41.66
		6000U	34.13
Vincristine	chemotherapy regimens	1.4 mg/m^2	39.21
Isophosphamide	chemotherapy regimens	2500 mg/m^2	32.34
		1200 mg/m^2	27
Dasatinib	molecular targeted therapeutic drugs	80 mg	32.31
Idarubicin	chemotherapy regimens	7 mg/m^2	31.27
		8 mg/m^2	26.72
Doxorubicin	chemotherapy regimens	60 mg/m^2	30.15
		40 mg/m^2	23.43
Mitoxantrone	chemotherapy regimens	14 mg/m^2	28.92
		8 mg/m^2	27.27
Decitabine	demethylation	15 mg/m^2	25.42
		20 mg/m^2	15.83
Pirarubicin	chemotherapy regimens	40 mg/m^2	22.79
		20 mg/m^2	<1
Cyclophosphamide	chemotherapy regimens	15mg/Kg	18.23
		10mg/Kg	16.55
Imatinib	molecular targeted therapeutic drugs	300 mg	7.76
Nilotinib	molecular targeted therapeutic drugs	230 mg	4.46
Methylprednisolone	glucocorticoid	40 mg	<1
Methotrexate	chemotherapy regimens	10 mg	<1
Rituximab	antibody	375mg/m^2	<1
